# Demyelination and Na^+^ Channel Redistribution Underlie Auditory and Vestibular Dysfunction in PMP22-Null Mice

**DOI:** 10.1523/ENEURO.0462-23.2023

**Published:** 2024-02-09

**Authors:** Jeong Han Lee, Seojin Park, Maria C. Perez-Flores, Yingying Chen, Mincheol Kang, Jinsil Choi, Lauren Levine, Michael Anne Gratton, Jie Zhao, Lucia Notterpek, Ebenezer N. Yamoah

**Affiliations:** ^1^Department of Physiology and Cell Biology, School of Medicine, University of Nevada, Reno, Reno 89557, Nevada; ^2^Prestige BioPharma, Busan 67264, South Korea; ^3^Program in Audiology and Communication Sciences, Washington University, St. Louis 63110, Missouri; ^4^Boys Town National Research Hospital, Omaha 68131, Nebraska

**Keywords:** demylination, hearing loss, neuropathy, PMP22, sodium channels, vestibular hypofunction

## Abstract

Altered expression of peripheral myelin protein 22 (PMP22) results in demyelinating peripheral neuropathy. PMP22 exhibits a highly restricted tissue distribution with marked expression in the myelinating Schwann cells of peripheral nerves. Auditory and vestibular Schwann cells and the afferent neurons also express PMP22, suggesting a unique role in hearing and balancing. Indeed, neuropathic patients diagnosed with PMP22-linked hereditary neuropathies often present with auditory and balance deficits, an understudied clinical complication. To investigate the mechanism by which abnormal expression of PMP22 may cause auditory and vestibular deficits, we studied gene-targeted *PMP22*-null mice. *PMP22*-null mice exhibit an unsteady gait, have difficulty maintaining balance, and live for only ∼3–5 weeks relative to unaffected littermates. Histological analysis of the inner ear revealed reduced auditory and vestibular afferent nerve myelination and profound Na^+^ channel redistribution without PMP22. Yet, Na^+^ current density was unaltered, in stark contrast to increased K^+^ current density. Atypical postsynaptic densities and a range of neuronal abnormalities in the organ of Corti were also identified. Analyses of auditory brainstem responses (ABRs) and vestibular sensory-evoked potential (VsEP) revealed that *PMP22*-null mice had auditory and vestibular hypofunction. These results demonstrate that PMP22 is required for hearing and balance, and the protein is indispensable for the formation and maintenance of myelin in the peripheral arm of the eighth nerve. Our findings indicate that myelin abnormalities and altered signal propagation in the peripheral arm of the auditory nerve are likely causes of auditory deficits in patients with PMP22-linked neuropathies.

## Significance Statement

Hearing loss and vestibular hypofunction are the most common sensory disorders. Patients diagnosed with PMP22-linked neuropathies, such as Charcot–Marie–Tooth (CMT) disease, often have the initial presentation in auditory and balance deficits. Using the PMP22-null mouse model, we show that the auditory and vestibular hypofunction ensues from auditory and vestibular neuron demyelination and Na channel abnormalities.

## Introduction

Hereditary peripheral neuropathies (HPNs) represent a heterogeneous group of neurological disorders characterized by proprioceptive sensory loss, ataxia, muscle weakness, and postural instability (Wilmshurst and Ouvrier, 2011). It is generally considered a peripheral neuropathy of the lower extremities, although rare trigeminal neuropathy and facial palsy case reports are indicated (Coffey and Fromm, 1991). Clinical studies also show loss of sound segregation, hearing, and vestibular impairment are constant presentations in the disease progression (Kovach et al., 2002) and, at times, the first chief complaint (Seeman et al., 2004). Demyelination of the eighth nerve in HPNs, including Charcot–Marie–Tooth diseases (CMTs), has been reported in some cases (Starr et al., 2003; Rance et al., 2012; Poretti et al., 2013), but in others it was less remarkable relative to the norm. The underlying mechanisms for the auditory and vestibular hypofunction in CMTs are unknown, albeit early presentations (Seeman et al., 2004). However, traces of the auditory and balance deficit may help in diagnosis and potential early intervention.

The eighth nerve subserves the gateway for the sound–balance–brain interface in the inner ear. It houses the cochlea and vestibular end organs, comprising hair cells (HCs) that encode sound, gravity, and head acceleration information by transducing mechanical stimuli into neural codes in action potentials (APs) of afferent neurons to the brain (Hudspeth, 1989). Approximately 90% of the auditory neurons (type I afferent neurons) are bipolar neurons, extending myelinated unbranched peripheral projections to inner hair cells (IHCs; Zhang and Coate, 2017) and central axons to the brain. Schwann cells seed myelin on the peripheral neurites, and oligodendrocytes myelinate the central axons. A transition zone between Schwann cells and oligodendrocyte-derived myelin has been identified (Toesca, 1996). At the axon initial segment (AIS), which is classically a dendrite for primary auditory neurons, there is a noncanonical zone called heminode (Liberman, 1980) that extends ∼30 µm from the IHC–auditory nerve (AN) synapse and serves as the AP initiation site (Hossain et al., 2005; Kim and Rutherford, 2016). Similar AP-initiating zones have been identified in vestibular afferent neurons (VANs; Chimento et al., 1994). The heminodes and nodes of Ranvier are decorated with Na^+^ channel clusters (Colbert and Pan, 2002; Hossain et al., 2005; Kim and Rutherford, 2016) to mediate AP initiation and saltatory propagation, ensuring swift acoustic and vestibular conduction (Huxley and Stampfli, 1949; Ritchie and Rogart, 1977; Browne et al., 2017). The distinct separation of the myelination machinery of auditory and vestibular neurons makes them ideal models for studying disease mechanisms with differential central and peripheral segments, as in peripheral neuropathies.

The *PMP22* gene encodes a 22 kDa glycoprotein and is expressed primarily by myelinated Schwann cells. PMP22 constitutes a critical component of peripheral nerve myelin, underpinned by multiple HPNs stemming from the allele's overexpression, deletion, and point mutations. Indeed, PMP22-linked neuropathies account for ∼70% of HPNs (DiVincenzo et al., 2014). PMP22-linked neuropathies are classified as type 1 HPNs, with the primary lesion being demyelination, but axonopathy and distal neurodegeneration frequently accompany the disease. In humans, the autosomal dominantly inherited CMT1A is most frequently associated with duplication of the intact *PMP22* gene (Szigeti and Lupski, 2009). In a smaller group of CMT type 1E patients, single amino acid substitutions in PMP22 have been reported (Huang et al., 2013). Haploinsufficiency of *PMP22* causes hereditary neuropathy with pressure palsy (HNPP) and compression-induced neuropathy (Attarian et al., 2020). The varied symptoms and presentations abound in auditory and vestibular hypofunction in CMT patients. In some clinical settings, profound hearing loss is seen early (Seeman et al., 2004); in others, it is progressive with no known mechanisms (Kovach et al., 2002; Giuliani et al., 2019). Although *PMP22*-linked monogenic human neuropathies are clear, the protein's function is not fully understood. To begin to investigate the underlying mechanism for the inner ear pathobiology of *PMP22*-linked disorders, we examined *PMP22*-deficient, complete knock-out (KO) mice, whereby the coding exons are replaced with the *β-galactosidase* reporter gene (Amici et al., 2006).

We report that the null deletion of *PMP22* results in profound auditory and vestibular hypofunction, with the hard-of-hearing features encompassing hidden hearing loss (HHL) and hearing loss (HL; Sergeyenko et al., 2013; Liberman, 2015). The balancing threshold to linear acceleration is severely compromised in *PMP22^−/−^* mice. The profound demyelination of the peripheral branch of the eighth nerve, Na^+^ channel clusters redistribution, but not Na^+^ current density, and increased K^+^ current density subserve the mechanisms for the observed auditory and vestibular deficits.

## Materials and Methods

### PMP22-deficient LacZ Mice

All procedures were performed under research guidelines of the Institutional Animal Care and Use Committee of the University of Nevada, Reno. Mice of either sex were studied using similar numbers. Transgenic PMP22-deficient LacZ mice were generated using the *VelociGene* targeting technology (Valenzuela et al., 2003), as previously reported (Amici et al., 2006). Heterozygous PMP22-deficient mice on the 129S1/SclmJ background were bred to generate *PMP22^+/+^*, *PMP22^+/−^*, and *PMP22^−/−^* pups. Genotype was determined by PCR using genomic DNA isolated from tail biopsies (Lee et al., 2014). All experiments in this study were done with 3–4-week-old littermates.

### ABR and distortion product otoacoustic emission measurements

*PMP22* mice (*PMP22^+/+^*, *PMP22^+/−^*, and *PMP22^−/−^*) littermates were tested at 3–4 weeks of age. Mice were anesthetized using ketamine (40 mg/kg) and xylazine (10 mg/kg) intraperitoneal injection. The body temperature was monitored using a rectal probe and maintained at 36.8 ± 1.0°C with a homeothermic device (Harvard Apparatus). ABR and distortion product otoacoustic emission (DPOAE) measurements were described previously (Dou et al., 2004). For ABR assays, thresholds were obtained by presenting tone bursts at 4, 8, 16, and 32 kHz and a clicking sound from 0 to 90 dB sound pressure levels (SPL) in 5 dB intervals. Tones were 2.5 ms, while click was 0.1 ms in duration, with a repetition rate of 21/s. Electrodes were placed subdermally behind the tested ear (reference), the vertex (active), and the back (ground). Evoked potentials were averaged over 512 repetitions and collected using a Tucker-Davis Technologies (TDT) RZ6 processor and BioSigRZ software. The threshold was defined as the lowest intensity of stimulation that yielded a repeatable waveform based on an identifiable ABR wave. To compensate for the base-to-apex sound propagation through the cochlea (von Bekesy, 1970) and accurately estimate auditory processing delays, we examined the latency between peaks I and II of the ABR using chirp sound stimulus (3–80 kHz, 3.5 ms duration; Benichoux et al., 2018).

DPOAE measurements were performed using the same TDT system with two calibrated MF1 speakers connected to an ER10B + microphone. Data was collected every 21 ms and averaged 512 times. DPOAEs were recorded using two pure tones with frequencies f1 and f2, using an f2/f1 ratio 1.2. Input/output (I/O) functions were obtained by increasing the primary tone L1 (and corresponding L2) in 5 dB steps from 20 to 80 dB SPL at 8, 16, and 32 kHz frequencies. During DPOAE testing, the probe assembly was placed in the mouse's left ear canal after visual inspection to ensure no ear infection or inflammation of the tympanic membrane. DPOAE thresholds were defined as the lowest level of f1 required to produce a DPOAE greater than −5 dB SPL (Chen et al., 2021).

### VsEP measurement

Mice were anesthetized using ketamine (40 mg/kg) and xylazine (10 mg/kg), and VsEP recordings were made, as described (Jones et al., 1999). The skull was stabilized with a head mount. Stimuli were linear acceleration pulses of 2 ms duration, nine pulses per second, presented in standard and inverted directions. Normal polarity was defined as the upward displacement of the shaker platform while inverted in the downward platform displacement. Stimulus amplitude was measured in jerk (da/dt, i.e., g/ms, where 1.0 g = 9.8 m/s^2^ and 1.0 g/ms = 9.8 µm/ms) using a calibrated accelerometer attached to the shaker platform. Stimulus amplitude ranged from −18 to +6 dB re 1.0 g/ms, adjusted in 3 dB steps. Two-channel signal averaging was used to resolve vestibular responses from background electroencephalographic activity. The electrophysiological activity was amplified (200,000×) and filtered (300 to 3,000 Hz), and VsEPs were recorded to normal and inverted stimulus polarities (1,024 points, 10 µs per point, 128 responses per averaged waveform). Three response parameters were quantified: threshold, peak latencies, and peak-to-peak amplitudes. The threshold is measured in dB re 1.0 g/ms and is defined as the stimulus amplitude midway between that which produced a discernible VsEP and that which failed to produce a response. The threshold measures the sensitivity of the gravity receptor end organs, utricle, and saccule. Thresholds, latencies, and amplitudes were averaged for each group and analyzed.

### Inner ear histological analyses of hair cells and synaptic counts

The cochlea was microdissected into three to four pieces, as described (Montgomery and Cox, 2016). Cochlear pieces were measured, and a frequency map was computed based on a 3D reconstruction of the sensory epithelium for HCs and synapse count of associated structures to relevant frequency regions using a custom plug-in to ImageJ (Muniak et al., 2013). Confocal *z*-stacks of the 4, 8, 16, and 32 kHz areas were collected using a Leica Stellaris 8 (Leica) and Nikon A1R laser scanning confocal microscope (Nikon Instruments). Images were gathered in a 1,024 × 1,024 raster using a high-resolution oil immersion objective (60× and 100×). IHCs at the frequency locations were quantified using myosin-VIIa–positive as an HC marker within a 70–100 μm field (Chen et al., 2021). Synaptic ribbons and postsynaptic markers could be counted manually using 3D (*x*–*y*–*z* axis) representations of each confocal *z*-stack with the microscopic image analysis software Imaris (Oxford Instruments).

The cochleae were intra-labyrinthine perfused through the oval and round windows with 4% paraformaldehyde (PFA) in phosphate-buffered saline (PBS). The samples were decalcified in 10% ethylenediaminetetraacetic acid (EDTA) in PBS for up to 48 h at 4°C. Microdissected pieces were immunostained with antibodies to the following: (1) mouse anti-C-terminal binding protein 2 (presynaptic marker, BD Biosciences, 1:200); (2) rabbit anti-myosin VIIa (HC marker, Proteus Biosciences, 1:600); (3) rabbit anti-Homer 1 (postsynaptic marker, Synaptic Systems, 1:250); (4) rabbit anti-Tuj1 (BioLegend, 1:500), chicken anti-Tuj1 (Abcam,1:500), and mouse anti-Tuj1 (Abcam, 1:500); (5) rabbit anti-PMP22 (Abcam, 1:500); (6) chicken anti-MBP (EnCor Biotechnology, 1:500), rabbit anti-MBP (Abcam, 1:500), mouse IgG2b anti-MBP (Abcam, 1:500); (7) chicken anti-MPZ (Abcam, 1:500); (8) goat anti-calretinin (Swant, 1:500); (9) mouse anti-calretinin (MilliporeSigma, 1:200); and (10) rabbit anti-calbindin (Cell Signaling Technology, 1:200) with appropriate secondary antibodies coupled to Alexa 405, 488, 568, and 647 fluorophores from Jackson ImmunoResearch and Thermo Fisher Scientific. DAPI labeled the cell nucleus after secondary antibody incubation. Samples were stained with phalloidin and mounted with Fluoro-Gel (Electron Microscopy Sciences). Images were captured under a confocal microscope.

### Electron microscopy

Cochlear sensory epithelia transmission electron microscopy (TEM) was performed as described previously (Nie et al., 2005). Three-week-old PMP22-null mice and the littermates were killed, and the cochleae were fixed in 2.5% glutaraldehyde in 0.1 M cacodylate buffer at 4°C overnight. After several washes with buffer alone, the cochleae were fixed in 1% osmium tetroxide at room temperature (RT) for 1 h. After that, the fixed cochleae were decalcified in 10% EDTA for 3–4 d. Fixed and decalcified cochleae were dehydrated using a graded ethanol series and embedded in epoxy resin. Ultrathin sections were cut with a diamond knife. Specimens were examined using an electron microscope as described (Nie et al., 2005).

### Electrophysiology

Spiral ganglion neurons (SGNs) were isolated from the mice's (*PMP22^+/+^* and *PMP22^−/−^*) inner ear using a combination of enzymatic and mechanical procedures (Lv et al., 2012). Male and female (3 weeks) mice were killed, and the temporal bones were removed and incubated in a solution containing Minimum Essential Medium with HBSS (Invitrogen), 0.2 g/L kynurenic acid, 10 mM MgCl_2_, 2% fetal bovine serum (FBS; v/v), and glucose (6 g/L). As previously described, the SGN tissue was dissected and split into apical and basal segments across the modiolar axis (Lv et al., 2010). The tissues were digested separately in an enzyme mixture containing collagenase type I (1 mg/ml) and DNase (1 mg/ml) in a water bath with a vibration rate of 70/min at 37°C for 15 min. After a series of gentle trituration in a centrifugation solution containing Minimum Essential Medium with HBSS, 0.2 g/L kynurenic acid, 10 mM MgCls, 10% PBS, and glucose (6 g/L), the cell pellets were reconstituted in 500 μl of culture media [Neurobasal-A, supplemented with 2% B27 (v/v), 0.5 mM L-glutamine, 100 U/ml penicillin; Invitrogen]. The sample was filtered through a 40 μm cell strainer for additional cell culture and electrophysiological experiments. We cultured SGNs for ∼48–72 h to allow the detachment of Schwann cells from neuronal membrane surfaces.

For membrane voltage measurements in SGNs, the extracellular solution for most experiments contained the following (in mM): 145 NaCl, 6 KCl, 1 MgCl_2_, 0–2 CaCl_2_, 10 D-glucose, and 10 HEPES, at pH 7.3. For perforated patch experiments, the tips of the pipettes were filled with the internal solution containing the following (in mM): 150 KCl, 10 HEPES, and 10 D-glucose at pH 7.3. The pipettes were front-filled with the internal solution and back-filled with a similar solution containing 250 μg/ml amphotericin and 2 mM Ca^2+^. For current measurements in SGNs, recordings were obtained using the whole-cell configuration of the patch-clamp technique. Signals were amplified using an Axopatch 200B (Molecular Devices) amplifier and filtered (bandpass 2–10 kHz). Data was digitized using an analog-to-digital converter (Digidata 1322; Molecular Devices). Patch electrodes were pulled from borosilicate glass capillaries with a Flaming/Brown microelectrode puller (P97, Sutter Instrument) and fire-polished to a final resistance of ∼2–3 MΩ. The SGNs were then filled with the following internal solution in mM: 112 KCl, 2 MgCl_2_, 0.1 CaCl_2_, 5 MgATP, 0.5 Na_2_GTP, 1 EGTA or 10 BAPTA, and 10 HEPES, pH 7.35, with KOH. The external solution consisted of (in mM) 130 NaCl (or 5 NaCl and 125 NMGCl), 5 KCl, 1 MgCl_2_, 1 CaCl_2_, 10 D-glucose, and 10 HEPES, pH 7.4, with NaOH. The bath solution was constantly perfused at 1–2 ml/min. The liquid junction potentials were measured and adjusted as described (Rodriguez-Contreras and Yamoah, 2001). All recordings were obtained at RT (20–22°C).

### Data analyses

Data were analyzed using pClamp10.2 (Molecular Devices) and Origin8.1 (MicroCal software). All measurements were presented as mean ± SD, and Student's test was used to determine statistical significance (*p* < 0.05). Analyses of voltage- and current-clamp data followed that described in previous reports (Lv et al., 2010, 2012; Levic et al., 2011).

## Results

### Auditory and vestibular deficits after null deletion of PMP22

**Figure 1.**
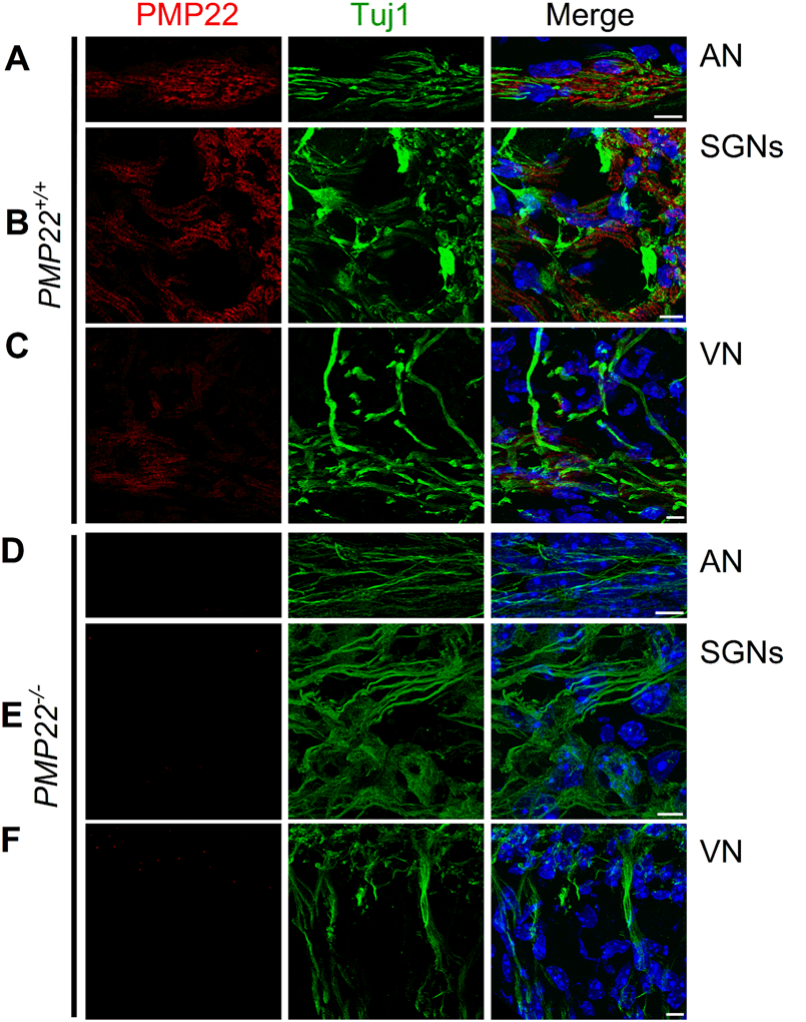
PMP22 expression in auditory and vestibular afferent neuron nerve fibers and effects of null deletion. ***A***–***C***, Immunofluorescent (IF) labeling of cochlear sections of wild-type mice (*PMP22^+/+^*) shows the expression of PMP22 (red). Neurons were labeled with Tuj1 (green). Scale bar, 10 µm. ***A***, ***B***, The panels below depict orthogonal sections of *PMP22^+/+^* mouse cochlear auditory nerve fibers (ANFs). ***C***, IF labeling of the vestibular ganglion's nerve fibers of wild-type mice (*PMP22^+/+^*) shows the expression of PMP22 (red). ***D***–***F***, In the PMP22-null (*PMP22^−/−^*) cochlea (AN and SGNs) and vestibule (VN), PMP22 labeling is absent. Scale bar, 10 µm.

**Figure 2.**
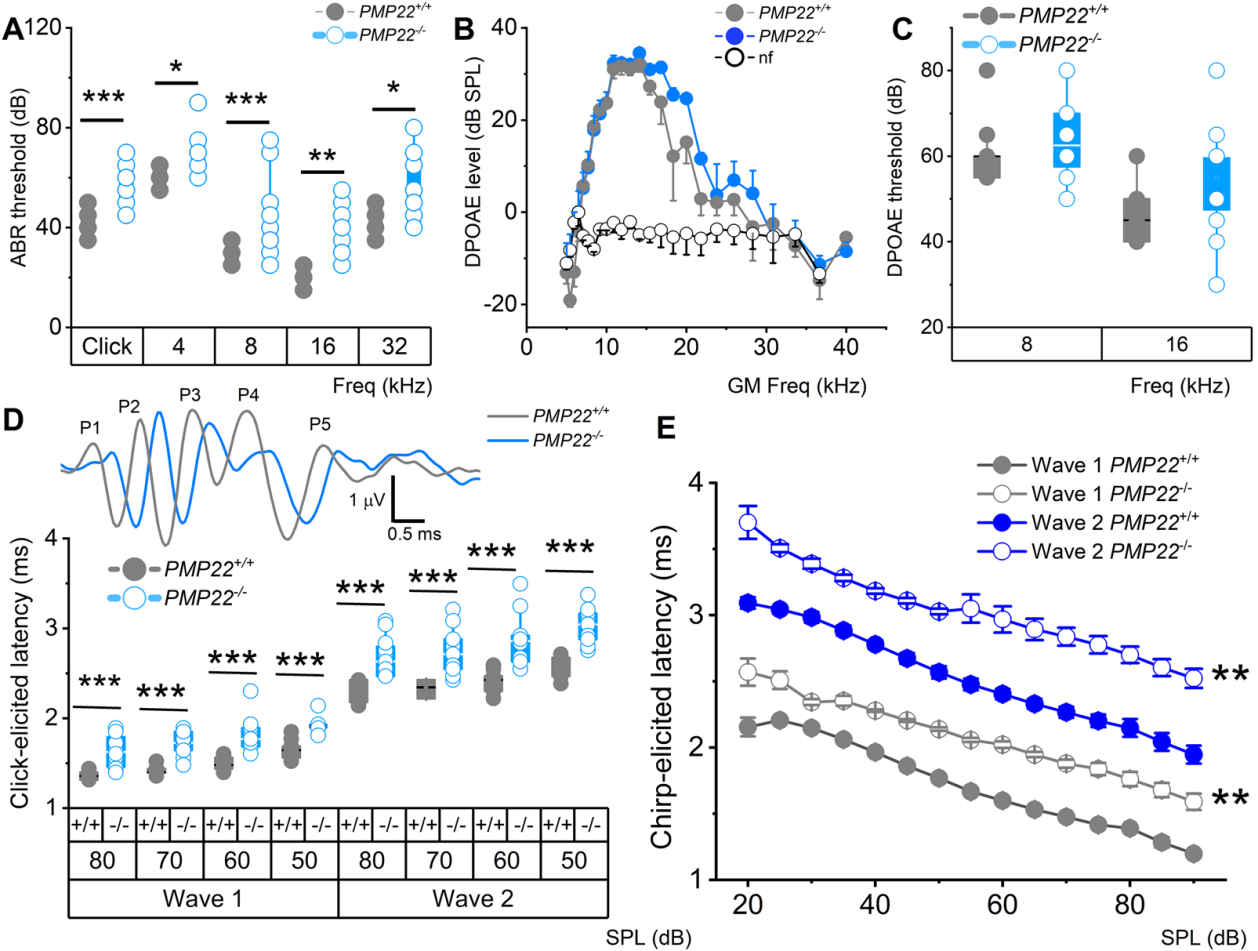
Assessment of auditory function in *PMP22^+/+^* and *PMP22^−/−^* mice. ***A***, ABRs were measured in 3-week-old *PMP22^+/+^* and *PMP22^−/−^* mice. Mean absolute ABR thresholds in response to clicking, tone pips at 4, 8, 16, and 32 kHz stimuli were significantly different between *PMP22^+/+^* and *PMP22^−/−^* mice. The mean values for ABR thresholds for sound stimuli were as follows (dB SPL): for a click, *PMP22^+/+^*^ ^= 42.7 ± 4.7 (*n* = 11); *PMP22^−/−^* = 57.1 ± 8.1 (*n* = 12); *p* = 4.3 × 10^−5^; for 4 kHz pip tone; *PMP22^+/+^* = 61.8 ± 4.1 (*n* = 11); *PMP22^−/−^* = 70.8 ± 10.0 (*n* = 12); *p* = 1.1 × 10^−2^; for 8 kHz pip tone; *PMP22^+/+^* = 29.6 ± 3.5 (*n* = 11); *PMP22^−/−^* = 44.2 ± 15.2 (*n* = 12); *p* = 9.5 × 10^−6^; for 16 kHz pip tone; *PMP22^+/+^* = 20.5 ± 3.5 (*n* = 11); *PMP22^−/−^* = 37.9 ± 9.4 (*n* = 12); *p* = 5.3 × 10^−3^; for 32 kHz pip tone; *PMP22^+/+^* = 43.6 ± 5.1 (*n* = 11); and *PMP22^−/−^* = 53.8 ± 14.0 (*n* = 12); *p* = 3.4 × 10^−2^. ***B***, DPgrams for *PMP22^+/+^* and *PMP22^−/−^* mice. The DPOAE levels for *PMP22^+/+^* (●) and *PMP22^−/−^* (●) mice are distinguishable and above the background [noise floor, shown with open circles (○)]. ***C***, Summary of DPOAE thresholds at 8 and 16 kHz. The mean values at 8 kHz were *PMP22^+/+^*^ ^= 60.5 ± 7.6 (*n* = 10); *PMP22^−/−^* = 64.2 ± 9.5 (*n* = 12); *p* = 3.4 × 10^−1^; and at 16 kHz were *PMP22^+/+^*^ ^= 45.9 ± 7.7 (*n* = 11); *PMP22^−/−^* = 55.0 ± 13.0 (*n* = 12); *p* = 6.0 × 10^−2^. ***D***, Inset shows ABR traces in response to 70 dB SPL sound clicks for *PMP22^+/+^* (gray) and *PMP22^−/−^* (light blue), and the characteristic five peaks (P1–5) are indicated. Average waves I and II latencies were determined for responses to sound clicks at 50, 60, 70, and 80 dB SPL. At all levels, the latency was prolonged (*n* = 12; *p* < 0.001). ***E***, Average waves I and II latency in response to chirp-sound at 20–90 dB SPL (mean ± SD) were significantly different at all levels (*n* = 11; *p* < 0.01).

Results from assessment of PMP22 expression in wild-type mice (*PMP22^+/+^*) inner ear revealed robust expression on the eighth nerve fibers of the auditory and vestibular systems (Fig. 1). The PMP22-null mutant's (*PMP22^−/−^*) eighth nerve were not only devoid of PMP22 expression but other myelin protein, for example, myelin basic protein (MBP), was often missing and when seen, was in atypical patchy clusters. We hypothesized that PMP22 is required for the eighth nerve myelination and expected its null deletion to alter the speed of auditory (sound) and balancing conduction and processing. Analyses of the absolute hearing threshold in response to clicking and pure tone chirps (4, 8, 16, and 32 kHz) showed a significant increase in sound levels (Fig. 2*A*). However, assessment of outer hair cell (OHC) functions by DPOAE detected no difference between the wild-type and the mutant mice (Fig. 2*B*,*C*). Results from the *PMP22^−/−^* mice had a striking resemblance to demyelination-mediated features of HHL (Liberman, 2015; Wan and Corfas, 2017). The sound conduction speed was monitored in ABR waves I and II latencies, reflecting IHC-to-SGN activity between *PMP22^+/+^* and *PMP22^−/−^* mice. The significant latencies recorded from click-sound–elicited waveforms among the two genotypes suggested conduction delay (Fig. 2*D*). We used chirp-sound–triggered ABR to account for the base-to-apex traveling wave delay. Chirp-sound–evoked waves I and II also yielded response latencies in keeping with the conduction delay (Fig. 2*E*).

**Figure 3.**
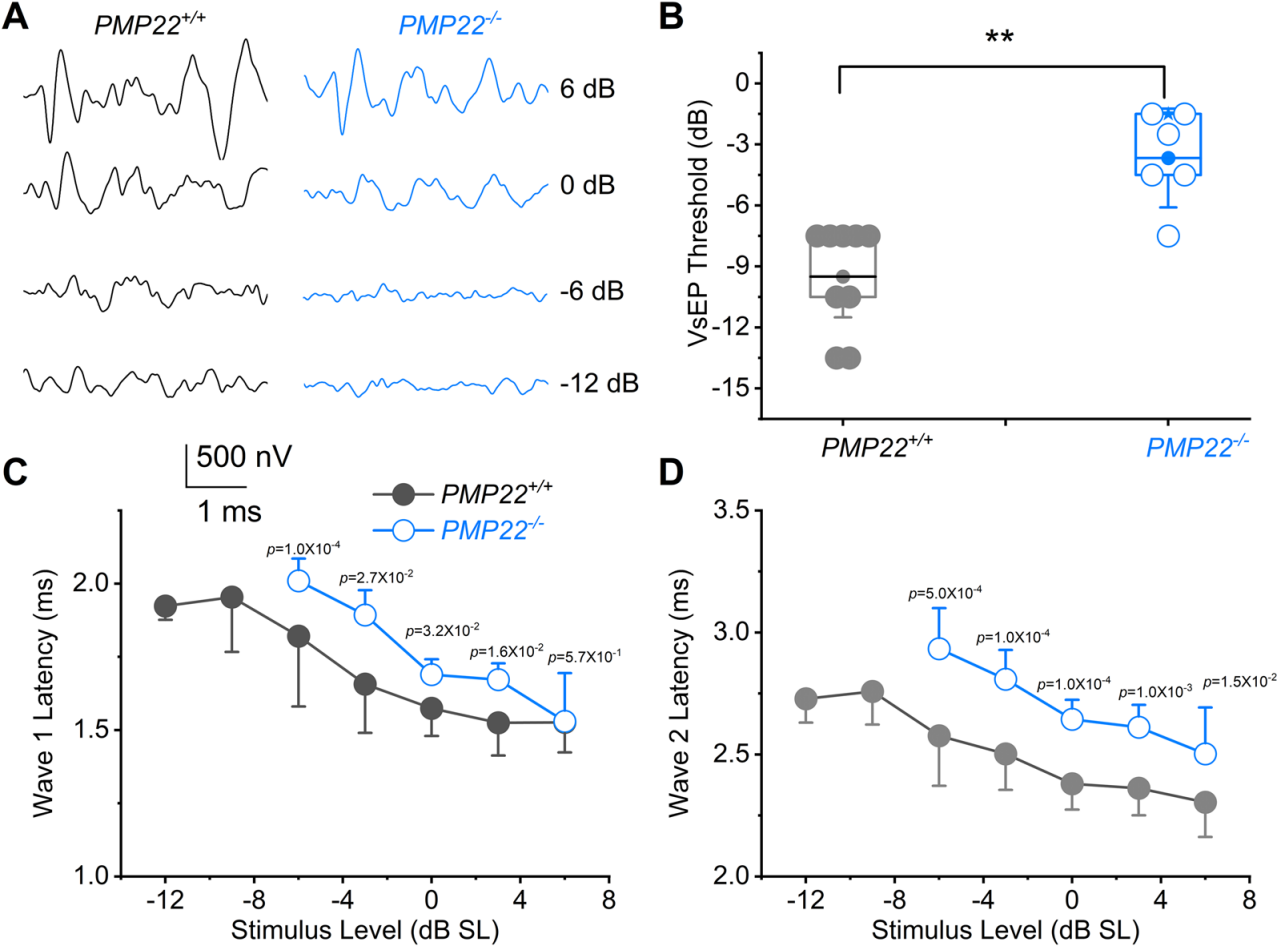
Vestibular function test for 3-week-old *PMP22^+/+^* and *PMP22^−/−^* mice. ***A***, Representative VsEP waveforms at different intensity levels are indicated. Stimulus intensities are shown in dB re 1.0 g/ms. ***B***, VsEP threshold for *PMP22^+/+^* (in gray) mice and PMP22^−/−^ (in light blue). Mean amplitudes are graphed (±SD). The thresholds for *PMP22^+/+^*, −9.5 ± 2.6 (*n* = 9) and *PMP22^−/−^*, −3.7 ± 2.5 (*n* = 6) are significantly different (*p* = 6.7 × 10^−4^). ***C***, ***D***, Mean latencies for waves I and II as an intensity function (*PMP22^+/+^*, *n* = 11 and *PMP22^−/−^*, *n* = 9).

The VAN sensitivity was assessed using linear VsEP measurements. Linear VsEPs are compound APs of the vestibular portion of the eighth nerve and its central relays (Jones and Jones, 1999; Jones et al., 2002; Zhao et al., 2008). They are generated by the utricle and saccule in response to linear jerk stimuli, with calyx-bearing afferents providing the most robust response. Profound deficits were evident in linear VsEPs recorded from *PMP22^−/−^*, relative to age-matched *PMP22^+/+^* mice (Fig. 3). The *PMP22^−/−^* mice had higher thresholds and delayed response peaks. The peak latencies of waves I and II were used for statistical analyses. Responses recorded from the left and right sides were similar, so the right side was chosen for study. Figure 3 shows the mean latencies as a function of stimulus intensities for −12 to 6 dB re 1 g/ms.

### Expression of myelin proteins in PMP22-null mutants

**Figure 4.**
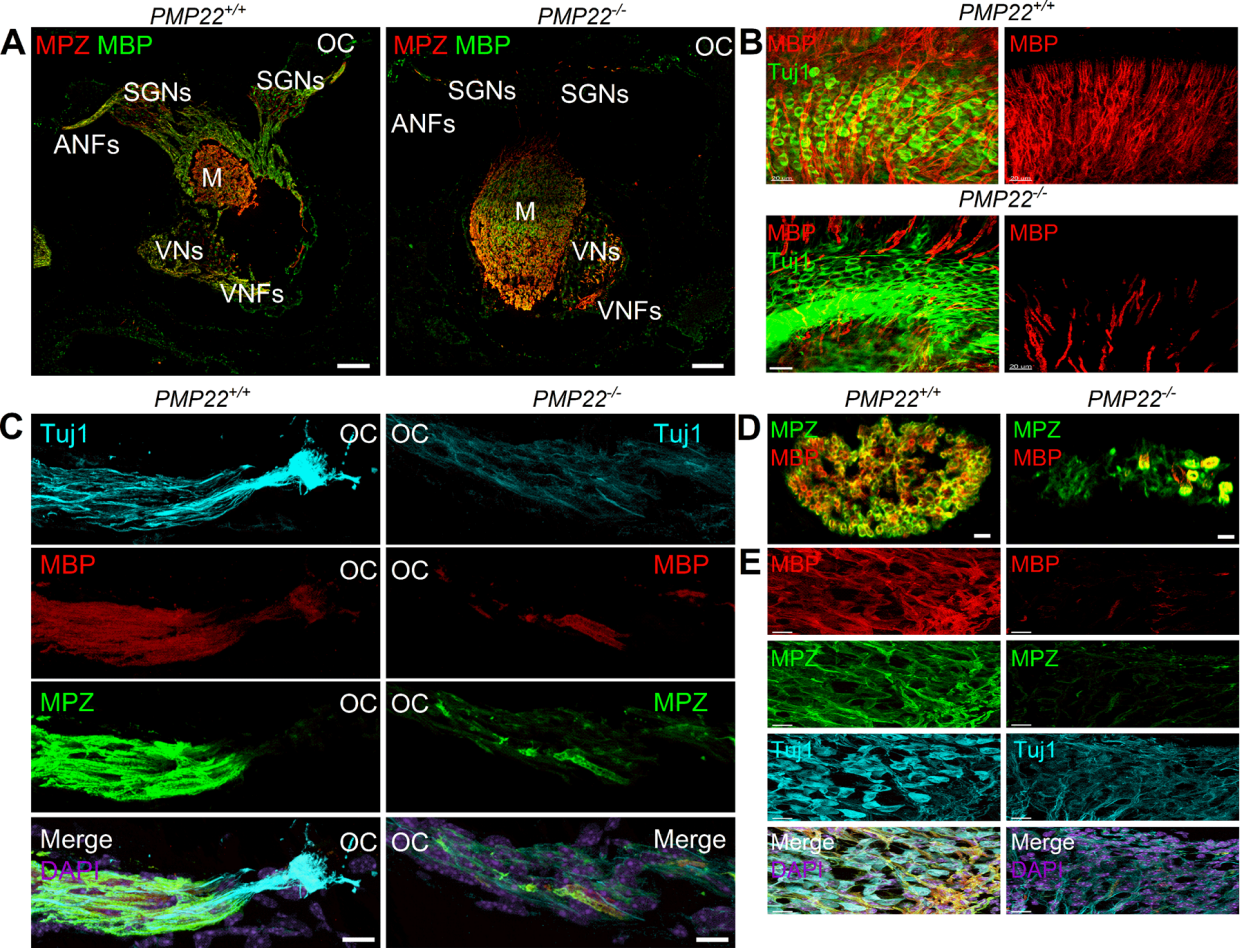
Compared with wild-type mice, reduced expression of MBP and MPZ in auditory and vestibular neurons in *PMP22^−/−^* cochlea. ***A***, Cross sections through the inner ear of *PMP22^+/+^* and *PMP22^−/−^* showing an overview of the organ of Corti (OC), SGNs, central ANFs, modiolar (M), vestibular neurons (VNs), and vestibular neuron fibers (VNFs). MPZ is in red, and MBP is in green. Scale bar, 100 µm. ***B***, SGNs were stained with Tuj1 (green), and the myelinated fibers were identified with MBP (red) antibodies. The top panels show images from *PMP22^+/+^* mice, while the bottom panels are the *PMP22^−/−^* cochlea. The neurites are devoid of MBP, but patches of MBP labeling can be seen. Scale bar, 20 µm. ***C***, A longitudinal section of the peripheral aspects of the auditory nerve was stained with Tuj1 (cyan), MBP (red), and MPZ (green). For orientation, the OC is indicated. Besides a few patches of MBP and MPZ expression in the *PMP22^−/−^* auditory nerve, they are devoid of myelin proteins compared with the *PMP22^+/+^*. Scale bar, 20 µm. ***D***, Sections through the ANFs from *PMP22^+/+^* and *PMP22^−/−^* mice are shown. Scale bar, 5 µm. ***E***, Sections through the VN reveal similar nerve fibers and neurons bare of myelin proteins. Scale bar, 10 µm.

**Figure 5.**
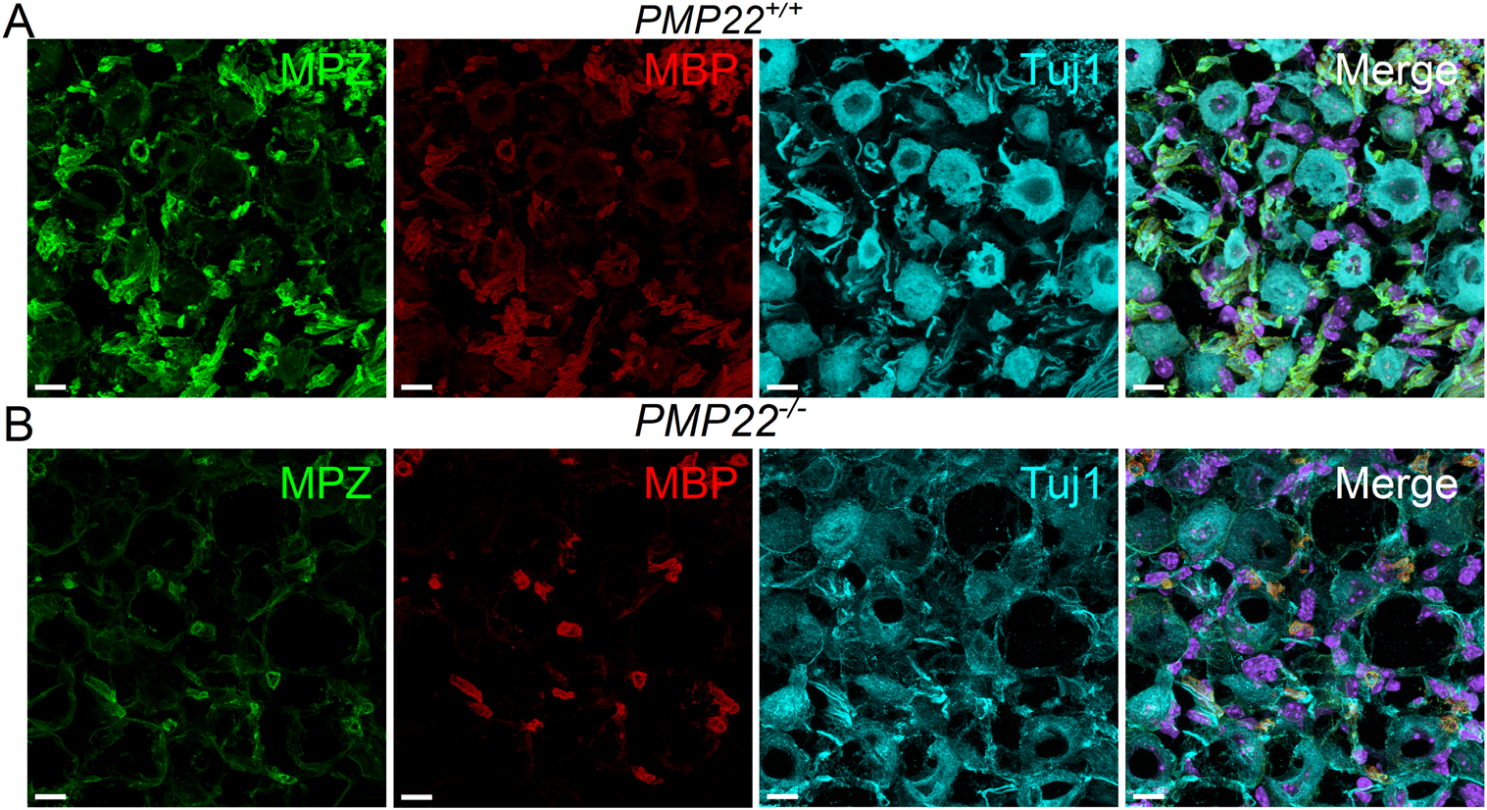
In PMP22^−/−^ vestibular neurons compared with *PMP22^+/+^* mice, MBP and MPZ expression is reduced. ***A***, ***B***, Cross section through the inner ear of *PMP22^+/+^* and *PMP22^−/−^* mice showing Scarpa ganglion vestibular neurons. MPZ is in green, and MBP is in red. Tuj1 staining serves as a neuronal marker, and DAPI is used for nuclear staining. Glial cells in the *PMP22^−/−^* mice display low levels of myelin proteins and, when expressed, appear condensed. Scale bar, 5 µm.

Myelin is a lipid-rich, multilamellar insulator of nerve fibers, with critical proteins underlying the molecular architecture of this specialized membrane (Arroyo and Scherer, 2000). Deletion of PMP22 could potentially disrupt the overall structure and prevent the formation of compact myelin (Amici et al., 2006). Therefore, we examined the expression of myelin protein zero (MPZ) and MBP in auditory and vestibular nerves in the inner ear of *PMP22^+/+^* and *PMP22^−/−^* littermates (Fig. 4). There was a robust expression of MPZ and MBP around nerve fibers in *PMP22^+/+^* cochlea and vestibule (Figs. 4, 5). In contrast, in the *PMP22^−/−^* mice, the distribution of MBP and MPZ was irregular and concentrated around a few fibers. A similar patchy expression of MPZ and MBP was observed around the cell bodies of VANs.

**Figure 6.**
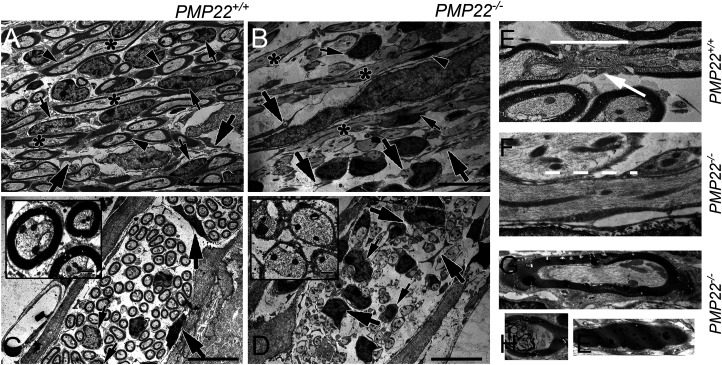
Lack of PMP22 results in loss of auditory (8th) nerve fibers and node of Ranvier. ***A***, An orthogonal view of the nodal region of the spiral laminaris in a *PMP22^+/+^* mouse shows that each eighth nerve fiber (asterisk*) is encased by myelin (arrowhead), produced by the Schwann cell (small arrow). A few macrophages (large arrow) are also noted, surrounding each nerve fiber. ***B***, A similar region from a *PMP22^−/−^* cochlea shows a loss of eighth nerve fibers (asterisk*), with myelin partially covering a single fiber (arrowhead). The number of satellite cells (small arrows) is diminished in the *PMP22^−/−^*, while macrophages (large arrows) are increased. ***C***, A cross-sectional eighth nerve bundle in the nodal region in a *PMP22^+/+^* mouse shows a typical neuronal packing density with a few large nucleated Schwann cells (small arrows) and nearby macrophages (large arrows). The inset illustrates the eighth nerve fiber's size range and the standard myelin sheath thickness. ***D***, Photomicrograph from *PMP22^−/−^* cochlea shows a loss of the eighth nerve fibers and satellite cells with increased macrophage activity. The diameter of the *PMP22^−/−^* fiber is unaffected by myelin loss. The higher-magnification insets reveal myelin loss is accompanied by phagocytotic activity as the macrophage surrounds and engulfs the fiber. ***E***, The nodal region of a *PMP22^+/+^* eighth nerve fiber shows myelin lamellae, particularly adjacent to the node (white solid bar and arrow). A thin layer of Schwann cell cytoplasm covers the node. ***F***, While a skinny layer of Schwann cell ensheathes the eighth nerve fibers (dashed white line), no defined nodes of Ranvier were detected in the *PMP22^−/−^* samples. ***G***, The presence of myelin surrounding the eighth fiber of a *PMP22^−/−^* cochlea was highly atypical. The myelin lacked the usual compact layers and contained very dark blobs, many of which had small lucent areas, thought to be the location of lipid, which was extracted during the tissue processing for TEM. ***H***, A second fiber is ensheathed by myelin, which contains amorphous, vacuolated dark material. ***I***, A giant blob of myelin was observed in a Schwann cell distant from any eighth nerve fibers. Scale bars: ***A–D***, 10 µm, inset, 500 nm; ***E–I***, 500 nm.

Ultrastructural analyses revealed the nodal region of the spiral laminaris in *PMP22^+/+^* mice of the eighth nerve fiber is encased by myelin produced by the Schwann cell, with only a few macrophages present (Fig. 6). In contrast, the norm for the *PMP22*-null cochlea was the loss of myelinated nerve fibers, and when present, thin myelin surrounded only a few fibers (Fig. 6). Meanwhile, the diameter of the *PMP22^−/−^* auditory nerve fibers relative to controls appeared unaffected, despite the lack of myelin (Fig. 6*C*,*D*). Schwann cell numbers were diminished in the *PMP22^−/−^* cochlea, while the macrophage numbers increased with phagocytotic activity. In the *PMP22^+/+^* mice, the nodal region of the eighth nerve fiber shows paranodal myelin loops adjacent to the node (Fig. 6*E*). Schwann cell microvilli cover the node, while a thin layer of the cytoplasm covers the myelin lamellae. The presence of myelin sheath in a *PMP22^−/−^* cochlea was unusual, with a collapsed appearance and no evidence of lamellae. Numerous small areas with lucent space consistent with myelin breakdown products were noted within the myelin sheath, ensheathed by myelin with a prominent tomacula, and sites of axonal myelin breakdown were apparent.

### Redistribution of Na^+^ channels and current density in PMP22-null mutant nerves

**Figure 7.**
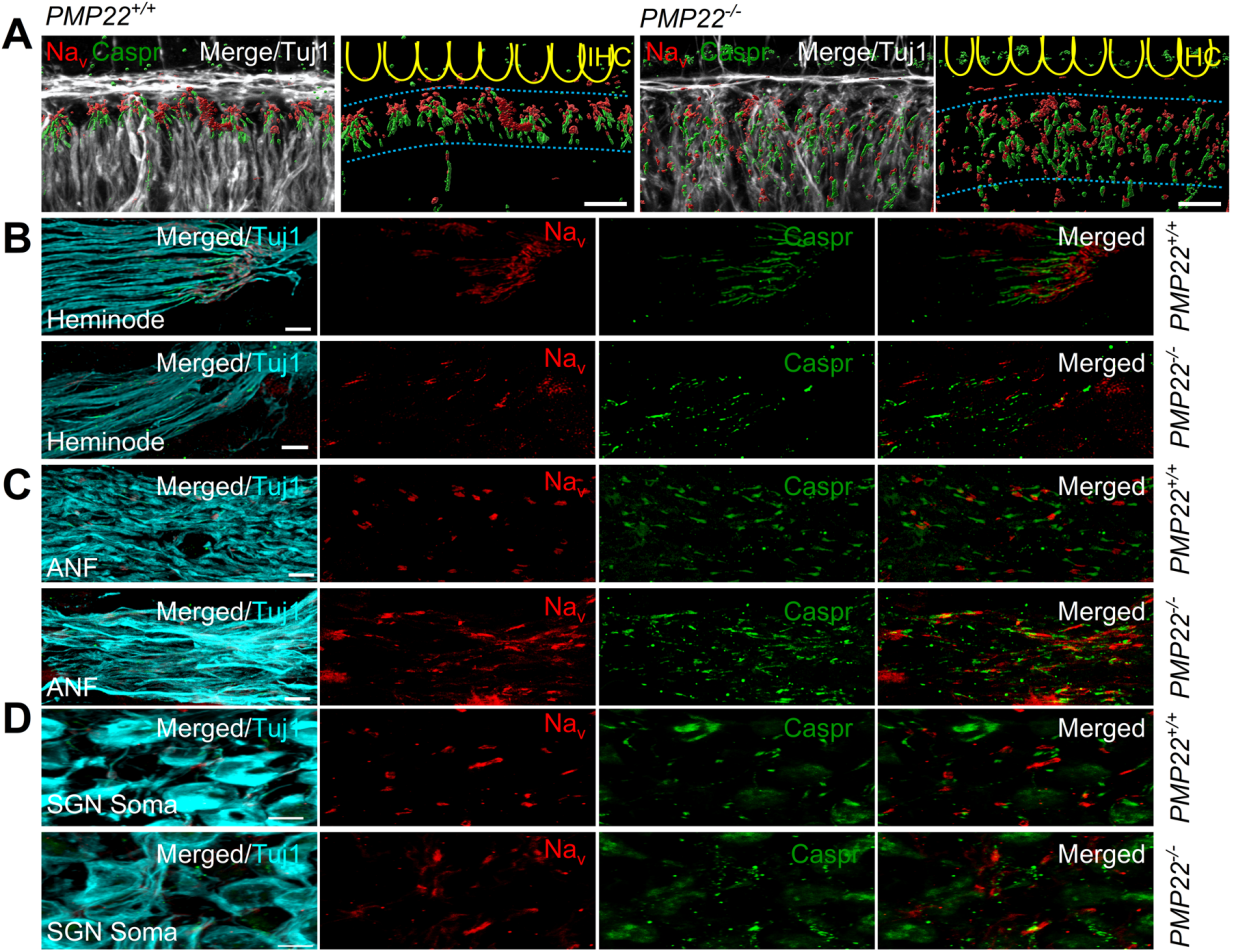
Na_v_ cluster distribution along the eighth nerve heminode and node of Ranvier. ***A***, ***B***, The image of the heminodal region of *PMP22^+/+^* mouse cochlea shows that the terminal is furnished with clustered sodium channels (Na_v_) in red. Caspr in green stains the paranodal regions. Tuj1 identifies (cyan) the eighth nerve fibers. Micrographs from *PMP22^+/+^* and *PMP22^−/−^* cochlea are indicated. ***C***, ***D***, Data showing the expression and distribution of Na_v_ along the ANFs and cell body (soma) of SGNs. Na_v_ is expressed but is distributed diffusely along the eighth nerve fibers (ANF). Scale bar, 10 µm. A longitudinal section along the eighth nerve exposes the nodes of Ranvier expressing Na_v_ clusters (in red) in *PMP22^+/+^* cochlea. Caspr in green stains the paranodal regions. Tuj1 stains (cyan) the eighth nerve fibers. The *PMP22^−/−^* cochleae have diffusely expressed Na_v_ (in red) along the eighth nerve fibers.

Clustered Na^+^ channels regulate the generation and propagation of APs at the AIS and at axonal nodes, to promote AP saltatory conduction. Because these sites are defined by myelin distribution, the disruption of myelin in the *PMP22^−/−^* nerve fibers raise the possibility that Na^+^ channels partitioning along auditory nerve fibers may be altered. The heminode (Liberman, 1980) is also a specialized segment of the auditory nerve decorated with increased Na^+^ channel density to serve as the AP initiating site (Hossain et al., 2005; Kim and Rutherford, 2016) for the forward-propagation of APs. In keeping with the typical Na^+^ channel expression, we observed clusters at the heminodes in *PMP22^+/+^* mice. Contrasting results were seen in *PMP22^−/−^* mice, where Na^+^ channels were expressed in a nonclustered and disorganized fashion along the nerve fiber (Fig. 7*A*,*B*). At the node of Ranvier, Na^+^ channels were distinctly clustered in *PMP22^+/+^* mice.

**Figure 8.**
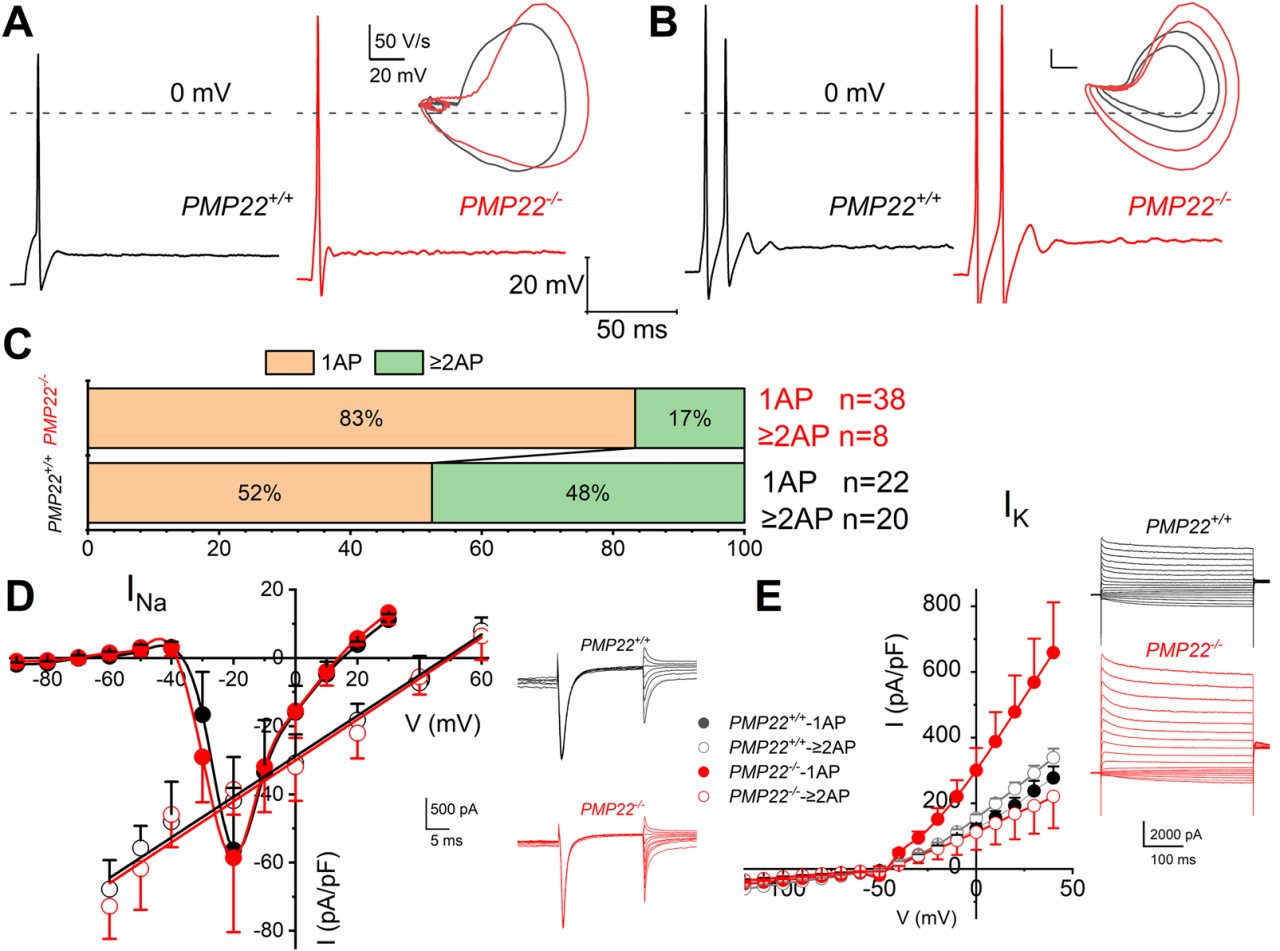
Changes in SGN AP features in *PMP22^+/+^* and *PMP22^−/−^* mice. ***A***, Representative AP traces recorded from *PMP22^+/+^* (in black) and *PMP22^−/−^* (in red) SGNs that respond to the current injection (0.1 nA) with a single AP from 6-week-old mice. The inset shows phase plots of the APs superimposed for comparison. AP threshold for the *PMP22^+/+^* [threshold = −43 ± 6 mV (*n* = 16) for 1-AP SGNs] was at membrane voltages ∼10 mV more negative than the *PMP22^−/−^* [threshold = −31 ± 3 mV (*n* = 11) for 1 AP SGNs], and wild-type SGNs were more excitable than the mutant. ***B***, Similar representation of SGNs that yield two or more APs in response to 0.1 nA current injection. Results from ***A*** and ***B*** have similar threshold profiles. ***C***, Summary, represented in the bar graph, of the shift in functional SGN population in *PMP22^−/−^* compared with the *PMP22^+/+^* mice. Among the SGNs sampled, there was a ∼30% increase in 1-AP SGNs in the mutant cochlea. ***D***, Current–voltage (I–V) of Na^+^ currents in SGNs generated from a holding voltage of −90 mV and stepped to depolarizing voltages with Δ*V* = 10 mV. Data from *PMP22^+/+^* are in black, and *PMP22^−/−^* SGNs are in red. The inset shows Na^+^ current traces used to generate the instantaneous current–voltage relationship fitted with a regression line. The reversal potential (*E*_rev_) of the Na^+^ current in *PMP22^+/+^* and *PMP22^−/−^* SGNs were 48 ± 4 and 51 ± 6 mV (*n* = 7), respectively. ***E***, Enhanced whole-cell K^+^ current in *PMP22^−/−^* SGNs eliciting 1-AP upon current injection. Voltage-clamp was recorded apical SGNs of 3-week-old *PMP22^+/+^* and *PMP22^−/−^* mice. SGNs were held at −70 mV and stepped from −110 to 40 mV using 10 mV increments (traces shown in the inset). Summary data of the current density–voltage (I/V) relationship indicates a significant increase in the current density of *PMP22^−/−^* SGNs at 3 weeks of age (***p = 0.001).

To examine the functional significance of Na^+^ channel redistribution and altered myelination on membrane properties in SGNs, we performed current-clamp recordings and studied membrane currents that may shape the AP profiles. The resting membrane potentials (*V*_rest_) appeared similar in SGNs from both genotypes [*PMP22^+/+^* = −62 ± 6 mV (*n* = 37); *PMP22^−/− ^*= −58 ± 8 mV (*n* = 29)]. SGNs isolated from *PMP22^+/+^* mice required less current injection (0.10 ± 0.05 nA; *n* = 28) to evoke an AP compared with SGNs from *PMP22^−/−^* mice (0.15 ± 0.07 nA; *n* = 25; Fig. 8*A*). Comparatively, AP elicited in *PMP22^+/+^* had a lower threshold than *PMP22^−/−^* SGNs. Similar *V*_rest_ and threshold voltage trends were obtained for SGNs that produced two or more APs upon current injection (Fig. 8*B*). Generally, *PMP22^−/−^* SGNs were less excitable relative to *PMP22^+/+^* with ∼30% shift toward 1-AP-elicited neuron (Fig. 8*C*).

To investigate the underlying mechanisms for the decreased membrane excitability in *PMP22^−/−^* SGNs, we performed whole-cell recordings on SGNs isolated from 3-week-old littermates. In the voltage-clamp configuration, when outward currents were suppressed with pipette Cs^+^, NMG^+^, and bath TEA, and inward Ca^2+^ currents curbed with bath-Ca^2+^ (0.1 mM), whole-cell currents revealed a transient inward current, mediated by voltage-dependent inward (Na^+^) current, that activated approximately −40 mV and peaked at approximately −20 mV. When activated with an instantaneous current-voltage protocol, the current reversed at 48 ± 4 (*n* = 7) and 51 ± 6 mV (*n* = 7) for the *PMP22^+/+^* and *PMP22^−/−^* SGNs, respectively. A summary of the current densities in the wild-type and mutant mice showed no quantitative differences (Fig. 8*D*). Because the observed membrane responses between the two genotypes could also be attributed to outward current alterations, we targeted outward K^+^ currents. Similarly, as described previously, inward currents were blocked to evaluate the magnitude of the whole-cell K^+^ currents. We identified a stark contrast between whole-cell outward K^+^ current amplitudes in SGNs of the *PMP22^−/−^* mice and their age-matched *PMP22^+/+^* controls (Fig. 8*E*), particularly in 1-AP elicited neurons. The precise identity of the K^+^ channel subtype/s altered in the SGNs would require future studies beyond the scope of the present report.

**Figure 9.**
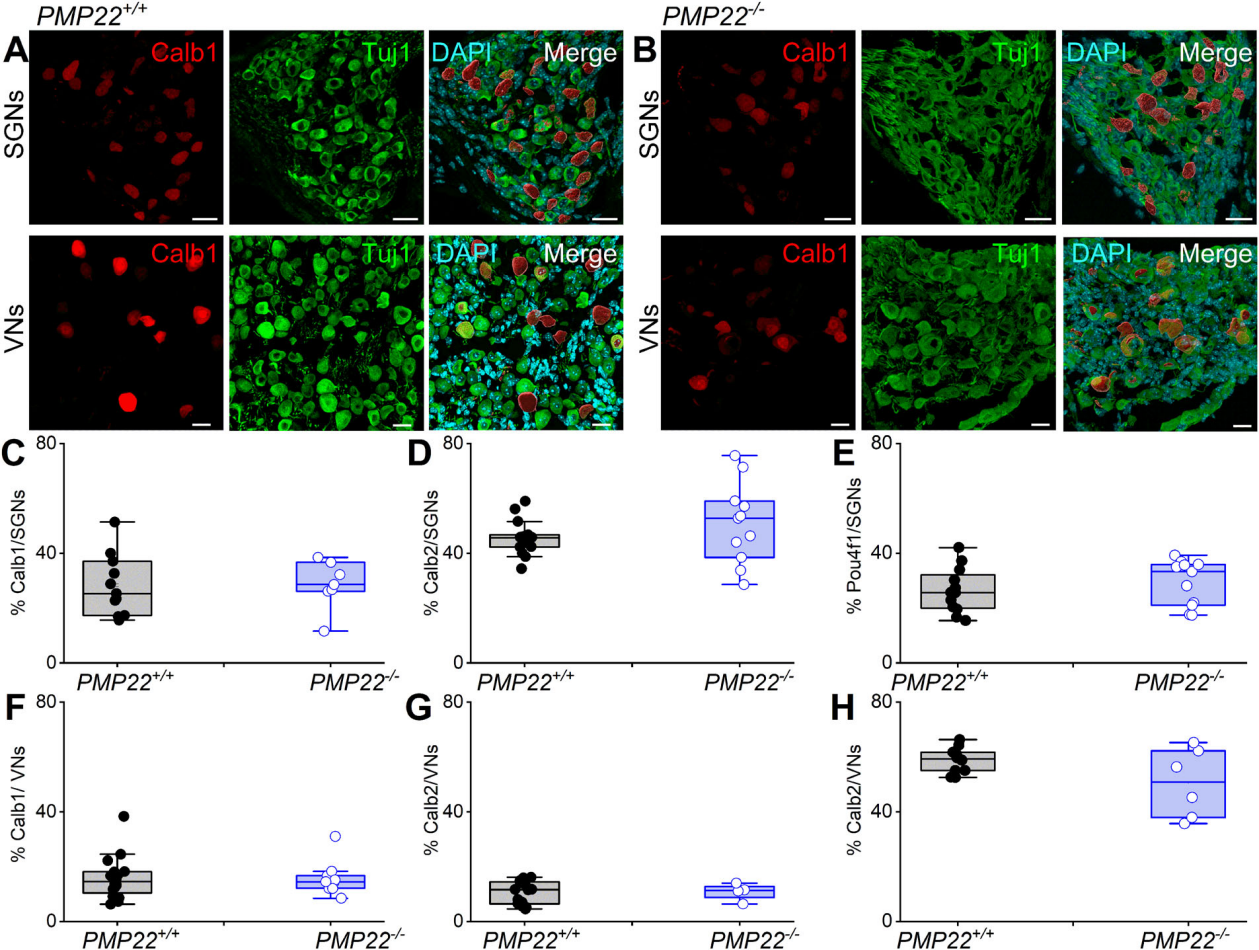
Afferent type I SGN subtype cell bodies are intact in 3-week-old *PMP22^−/−^* mice. ***A***, ***B***, Photomicrographs of a section through *PMP22^+/+^* and *PMP22^−/−^* inner ear show calbindin (Calb1)-positive neurons (red) in the spiral and vestibular ganglion (aka, Scarpa ganglion). Neurons were stained with Tuj1 (green). Scale bar, 10 µm. ***C****–**E***, Summary data of “blind” neuronal counts by three individuals. Similar analyses were performed for calretinin (Calb2) and Pou4f1-positive neurons. ***F***–***H***, Evaluation of Calb1-, Calb2-, and Pou4f1-positive VNs in the Scarpa ganglion. The two genotypes had no statistical differences in the indicated neuronal counts (*n* = 9; mean samples from 25 sections from 4 mice).

**Figure 10.**
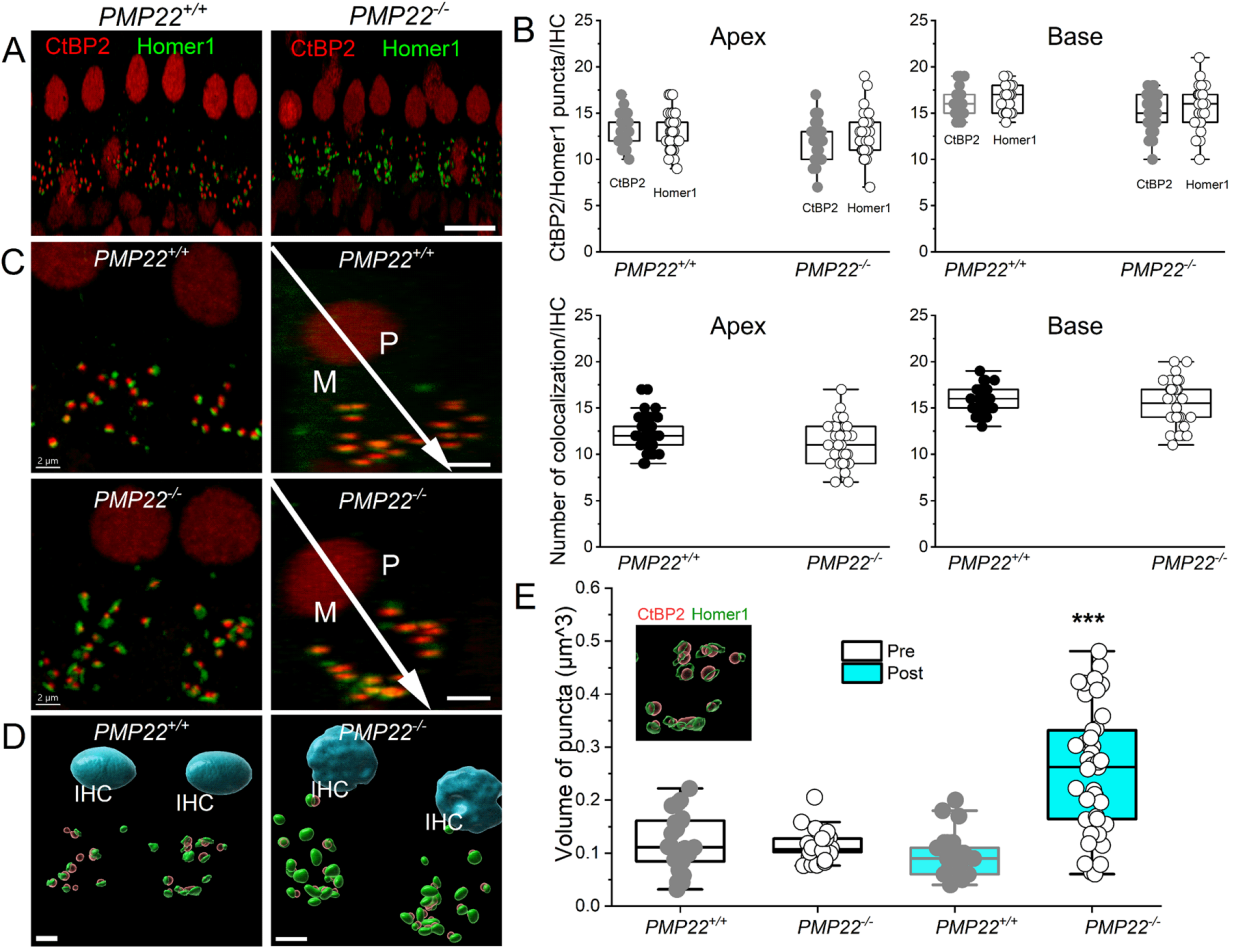
Presynaptic and postsynaptic marker counts are similar between *PMP22^+/+^* and *PMP22^−/−^* mice. Synapses between the SGNs and IHCs were quantified at two tonotopic locations [4–6 (apex) and 32–48 (base) kHz] in the organ of Corti isolated from 3-week-old *PMP22^+/+^* and *PMP22^−/−^* mice. ***A***–***D***, There were no obvious differences between *PMP22^+/+^* and *PMP22^−/−^* mice in the organization of afferent synapses, identified as paired CtBP2 (red) and Homer1 (green) immunopuncta. Images presented as *z*-projections were made using stacks of confocal micrographs from the apex and base of the cochlea. ***B***, Quantifying the average number of synapses per IHC shows no statistically significant differences between *PMP22^+/+^* and *PMP22^−/−^* mice (*n* = 8 from 4 mice). Analyses of colocalization of the pre- and postsynaptic markers revealed no quantitative differences. ***C***, *z*-stack projections of confocal micrographs from cochlear apex showing the modiolar (M) and pillar (P) aspects. ***D***, 3D enhancement of IHC synapse illustrating pre- and postsynaptic marker volume. ***E***, The synaptic puncta volume summary indicates a significant increase in postsynaptic volume in the *PMP22^−/−^* IHCs. Mean volume (in µm^3^) for *PMP22^+/+^* presynaptic puncta = 0.12 ± 0.06 (*n* = 10 from 5 mice); *PMP22^−/−^* presynaptic puncta = 0.11 ± 0.03 (*n* = 8 from 4 mice). Mean volume (in µm^3^) for *PMP22^+/+^* postsynaptic puncta = 0.10 ± 0.04 (*n* = 10 from 5 mice); *PMP22^−/−^* postsynaptic puncta = 0.26 ± 0.12 (*n* = 8 from 4 mice); comparing postsynaptic puncta, *p* = 2.2 × 10^−9^. The inset shows an enlarged micrograph of puncta volume. Values (mean ± SD) are illustrated (*p < 0.05; **p < 0.01; ***p < 0.001). Scale bars: ***A***, 5 µm; ***C***, ***D***, 2 µm.

The observed shift in membrane excitability in the *PMP22^−/−^* SGNs (Fig. 8*C*) raised the possibility of SGN subtype (Shrestha et al., 2018; Sun et al., 2018) alterations. No significant restructuring of neuronal subtypes was identified using calbindin, calretinin, and pou4f1 as markers for SGNs and VANs (Fig. 9). SGNs can show delayed loss after earlier loss of synaptic contacts to the IHCs. We examined the synaptic connections between the IHCs and SGNs at two tonotopic locations [4–8 (apex) and 32–45 (base) kHz] and found no observable qualitative differences between *PMP22^+/+^* and *PMP22^−/−^* mice in the organization of afferent synapses, identified as paired CtBP2 (red) and Homer1 (green) immunopuncta (Fig. 10*C*,*D*). Quantifying the mean number of synapses per IHC indicated no statistically significant differences between *PMP22^+/+^* and *PMP22^−/−^* mice when comparing tonotopic regions (Fig. 10*B*; *p* values = 0.9543 for apex and 0.8315 for base; ordinary one-way ANOVA with Sidak's correction for multiple comparisons). Thus, despite differences in the ABR wave I responses between the studied genotypes, which mimicked HHL in the *PMP22^−/−^* mice, there were no indications of changes in pre- and postsynaptic numbers in the cochleae of *PMP22^−/−^* mice. However, morphological changes in the postsynaptic marker volume in the *PMP22^−/−^* mice (Fig. 10*E*) implicate synaptopathy as a contributory factor in the documented HHL and HL.

## Discussion

The auditory and vestibular systems process the fastest time-limiting sensory codes and the swiftest human reflex (Huterer and Cullen, 2002). Among the essentials for speed is nonquantal neurotransmission in the calyceal synapse between vestibular HCs and first-order neurons (Eatock, 2018). A recently identified phenomenon wherein primary auditory neurons are directly, mechanically sensitive at the non-myelinated synaptic terminals may also shape the speed of auditory temporal coding (Perez-Flores et al., 2022). The peripheral dendrites of the eighth nerve, which subserve the sound–balance–brain interphase, are furnished with compact myelin at the internodes to reduce membrane capacitance and facilitate AP speed propagation. Major protein constituents of compact myelin are MPZ, MBP, and PMP22. Although PMP22 comprises ∼2–5% of the total proteins in myelin, its functional importance is underpinned by mutations of the *PMP22* allele that result in common forms of CMTs, a disease with an estimated prevalence of one in 2,500 people (Skre, 1974). The typical and dominant symptoms of CMT neuropathies are characteristic slowed nerve conduction velocity and diminished amplitudes of motor and sensory responses of the extremities (Dyck and Lambert, 1968). However, the condition is marked by expanding phenotypic heterogeneity, including hearing loss and balance defects being some of the early features of the disease (Seeman et al., 2004; Rance et al., 2012; Estilow et al., 2019). The near-instant reactiveness of the auditory and vestibular systems to stimuli makes them serve as gateways to sensory-deficit–prone diseases.

The objectives of the present study were to analyze the auditory and vestibular phenotype of *PMP22*-deficient mice to investigate the physiological functions of PMP22 in vivo. The homozygous mutant mice did not express detectable PMP22 protein, confirming the effectiveness of the null mutation strategy (Valenzuela et al., 2003). Homozygous mutants were born in the standard Mendelian ratio but exhibited excess mortality (mean live span ∼3–5 weeks), demonstrating that PMP22 is not essential for embryonic development but is required for survival after birth. Severe ataxia was evident after 2 weeks, and analyses of linear vestibular and auditory functions revealed balance deficit and hearing loss. Because the peripheral aspects of the eighth nerve myelination are conferred by Schwann cells, and PMP22 is a principal functional constituent of compact myelin (Amici et al., 2006), we considered the likelihood that the ataxia and hearing loss might emanate from the inner ear. It is conceivable that a component of the sensory and motor disorders at the lower extremities and cerebellar dysfunction may contribute to the ataxia phenotype but not the auditory system. Here, we focused on the inner ear abnormalities.

The sense of balance and equilibrium are detected by the vestibular end organs, namely, the utricle and saccule, which sense changes in linear acceleration and head position relative to gravity, and the semicircular canals, which detect changes in angular acceleration. The hair cells in epithelia detect the motion, and the myelinated afferent neurons establish giant calyceal and bouton synapses (Eatock, 2018) to convey neural codes to the brain. The neural signals are the difference in the discharge of the firing rate of vestibular afferents, modulating the tonic firing rate on both sides in opposite directions. Surface preparation and histological assessment of the three end organs of *PMP22^−/−^* mice did not reveal any abnormalities, nor did the gelatinous cupula within the ampulla of each duct and the otolithic membrane overlying the sensory epithelia. However, *PMP22*-null mutants’ afferents lacked compact myelin, and the expression of myelin proteins was sparse. A similar lack of compact myelin formation has been seen in the sciatic nerve of young mutant mice (Amici et al., 2006), supporting a role for PMP22 in postnatal nerve development. The fact that the null mutants exhibited difficulties maintaining their balance at a standstill and in motion and delayed VsEP, is consistent with a deficit in vestibular afferent AP discharge. Thus, the lack of myelin provides a striking histopathological correlation of the defect in balance.

Other striking observations were the hearing deficits and the histopathology of the auditory nerve fibers in *PMP22*-null mice. ABR measurements using click and chirp sound, accounting for sound traveling waves along the cochlear frequency axis, showed *PMP22^−/−^* mice had severe HHL, and threshold assessment revealed profound hard-of-hearing. Notably, the PMP22 transcript is known to be expressed in the human fetal cochlea (Robertson et al., 1994). Loss of auditory fibers, absence of compact myelin, lack of defined nodes of Ranvier, and subsequent loss of nodal Na^+^ channel clusters and heminodal Na^+^ channels, are abnormalities that likely curtail AP initiation and propagation, resulting in ABR and VsEP waves I and II latencies. The observed unaltered Na^+^ current densities in the *PMP22*-null mutants relative to the *PMP22^+/+^*, at least in the cell bodies of auditory and vestibular neurons, suggest that Na_v_ expression and membrane trafficking remain intact, but the channel localization mechanisms may be warped in the absence of PMP22. In contrast, neuronal demyelination diseases have been shown to increase K^+^ current (Ng et al., 2008), which is consistent with the present findings, although the exact mechanism remains unclear.

Schwann cells may be the recognized primary hub for PMP22 synthesis, but the protein expression has a sensory neuronal component regulating the signaling pathways in myelination (Maier et al., 2002, 2003). Sensory neurons with distinct regulatory elements to endorse the cross talk between their upstream promoters and Schwann cells may constitute important myelination components and subserve PMP22 mutation and disease pathology. Additionally, PMP22 may not be a mere component of the myelin protein complex but an important molecule for early neural development (Parmantier et al., 1997; Wulf et al., 1999). The severe disease phenotype and rare occurrence of neuropathic patients with PMP22-null genotypes argue for a broader role for PMP22 beyond myelin synthesis. Indeed, a case report of a severely affected PMP22-null boy described predominantly large fiber sensory loss, with decreased proprioception and sensory ataxia (Saporta et al., 2011). Sensorineural hearing impairment, with progression beyond presbyacusis, has also been reported in patients with heterozygous deletion of the PMP22 gene (Verhagen et al., 2005). While the current study did not include heterozygous PMP22-deficient mice, the described morphological and electrophysiological data provide novel insights into the requirement of PMP22 in the inner ear. Future studies will examine how other disease-linked PMP22 mutations impact auditory and vestibular systems.

## Synthesis

Reviewing Editor: Benjamin Deneen, Baylor College of Medicine Decisions are customarily a result of the Reviewing Editor and the peer reviewers coming together and discussing their recommendations until a consensus is reached. When revisions are invited, a fact-based synthesis statement explaining their decision and outlining what is needed to prepare a revision will be listed below. The following reviewer(s) agreed to reveal their identity: Ahmet Hoke. Note: If this manuscript was transferred from JNeurosci and a decision was made to accept the manuscript without peer review, a brief statement to this effect will instead be what is listed below.

This paper has been seen by two experts who both found the paper to be well done and of interest to the field. Reviewer 1 raises a few concerns about figure quality, particularly in Figure 1 (MBP staining) and Figure 7 (Nav staining). Please augment these figures prior to publication.
